# Identification of (2*R*,3*R*)-2-(3,4-dihydroxyphenyl)chroman-3-yl-3,4,5-trihydroxy benzoate as multiple inhibitors of SARS-CoV-2 targets; a systematic molecular modelling approach†

**DOI:** 10.1039/d1ra01603b

**Published:** 2021-04-07

**Authors:** Jubie Selvaraj, Shyam Sundar P, Logesh Rajan, Divakar Selvaraj, Dhanabal Palanisamy, Krishnan Namboori PK, Suresh Kumar Mohankumar

**Affiliations:** Department of Pharmaceutical Chemistry, JSS College of Pharmacy, JSS Academy of Higher Education & Research Rockland's, Ooty, Nilgiris 643001 Tamil Nadu India; TIFAC CORE in Herbal Drugs, Department of Pharmacognosy, JSS College of Pharmacy, JSS Academy of Higher Education & Research Rockland's, Ooty, Nilgiris 643001 Tamil Nadu India; Department of Pharmacology, JSS College of Pharmacy, JSS Academy of Higher Education & Research Rockland's, Ooty, Nilgiris 643001 Tamil Nadu India; Amrita Molecular Modelling and Synthesis (AMMAS) Research Lab, Amrita Vishwavidyapeetham Amrita Nagar, Ettimadai Coimbatore Tamil Nadu India; Swansea University Medical School, Swansea University Singleton Park Swansea Wales SA2 8PP UK s.k.mohankumar@swansea.ac.uk

## Abstract

Coronavirus disease of 2019 (COVID-19) is a zoonotic disease caused by a new severe acute respiratory syndrome (SARS-CoV-2) which has quickly resulted in a pandemic. Recent anti-COVID-19 drug discoveries are leaning towards repurposing phytochemicals which have been previously reported for SARS and MERS-CoV outbreaks. However, they have been either virtually screened or tested so far against mono targets and the potent derivatives of virtually sorted lead molecules remain elusive. We aimed to identify the phytochemicals having potentials to inhibit SARS CoV-2 infection *via* multiple targets. The selected 132 phytochemicals were virtually screened using a structure based *in silico* technique against main protease (M^pro^) which is a potential target of SARS CoV-2. Six compounds were selected based on the LibDock scores and further subjected to induced fit docking using the CDOCKER module of DS. Two compounds namely cinnamtannin-B and gallocatechin gallate were identified as top HITS against main protease (M^pro^). Based on the Lipinski rule of five (L-ROF) and synthetic feasibility, gallocatechin gallate was taken for our further studies. Six analogues of gallocatechin gallate were screened against the next important targets such as RNA-dependent RNA polymerase (RdRp), angiotensin converting enzyme-2 (ACE2), transmembrane protease serine -2 (TMPRSS2) and interleukin-6 (IL-6) along with main protease (M^pro^). Our molecular docking results reveal that a gallocatechin analogue (GC-2) namely (2*R*,3*R*)-2-(3,4-dihydroxyphenyl)chroman-3-yl-3,4,5-trihydroxy benzoate has shown potential to inhibit multiple targets of SARS CoV-2. Further, the molecular dynamics study was carried out to ascertain the stability of the GC-2 and RdRp complex.

## Abstract 2

Structure-based insilico-guided screening of potential polytargeting anti-COVID phytochemical: gallocatechin gallate analogue.

## Graphical abstract

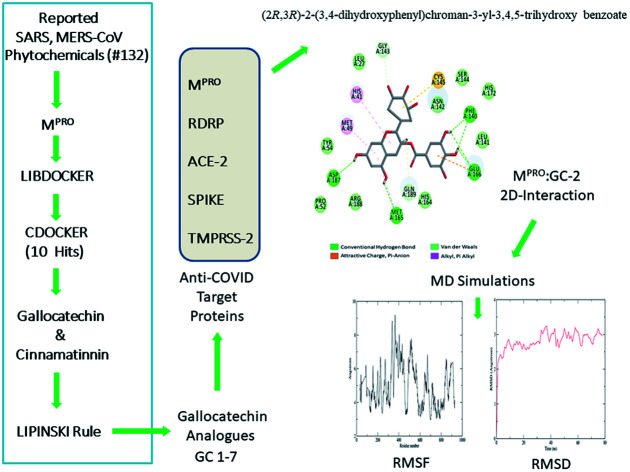

The novel coronavirus disease of 2019 (COVID-19) is an infectious disease and responsible for the 2019–2020 viral pneumonia outbreaks. The World Health Organization (WHO) declared that the outbreak of COVID-19 is pandemic on the 22^nd^ of April 2020. Sadly, this pandemic is still enduring and hence there is an urge to find novel prophylactic or therapeutic measures to combat the COVID-19 pandemic.^1–4^ However, to date there have been no specific therapeutics proven to eradicate COVID-19, which warrants multi-focused and multi-targeted strategies like, drug candidates having potentials to act on multiple antiviral targets and concurrently boost the host immune system.^5,6^ The US FDA has launched the Coronavirus Treatment Acceleration Program (CTAP) and specific guidance on COVID-19 clinical trials. Additionally, the FDA has issued emergency use authorizations for some drugs, for example, chloroquine, hydroxychloroquine, and Remdesivir. However, recent clinical trials investigating this drug concluded that the treatment was not significantly correlated to intubation risk or mortality by multiple analyses. Remdesivir, a nucleotide prodrug, inhibits viral RNA-dependent RNA polymerase and has been reported to have *in vitro* activity against SARS-CoV-2.^7^ There are now seven known coronaviruses that cause disease in humans to include, HCoV-229E, HCoV-OC43, HCoV-NL63, HCoV-HKU1, severe acute respiratory syndrome coronavirus (SARS-CoV) and Middle East respiratory virus coronavirus (MERS-CoV), and now SARS-CoV-2.^8^ The first four CoVs cause mild and self-limiting disease. However, the last three coronaviruses are highly pathogenic, leading to communicable outbreaks causing fatal respiratory diseases. Coronaviruses have the largest genomes among all known RNA viruses, ranging from 26 to 32 kb in length, which encode structural and nonstructural proteins. Several structural components of the coronavirus family viruses have been described as bio targets for drug discovery. These targets are related to viral nucleic acids, enzymes, spike glycoprotein, and envelope (membrane, nucleocapsid, and accessory proteins).^7^ A massive effort has been undertaken by the international scientific community to obtain structural information about complexes between SARS-CoV-2 targets and different types of inhibitors, providing information to use ligand-based (LB) and structure-based (SB) approaches to drug repurposing. The drugs such as amodiaquine and darunavir have also been repurposed for Covid therapy through *in silico* tools.^9^ There are also reports for the organic compounds such as alpha ketoamides and 3-alkynyl substituted 2-chloroquinoxaline frame work as lead compounds for Covid therapy.^10,11^ The present work evaluated the druggability of the SARS-CoV-2 main protease (SARS-CoV-2 M^pro^) as a potential target for the approved drug for the treatment of COVID-19 as it plays a crucial role in the cleavage of viral polyproteins involved in transcription and replication. Many research teams have exploited the major protease (M^pro^) of SARS-CoV-2, also named chymotrypsin-like protease (3CLpr),^12^ as a potential drug target to fight COVID-19. Sequence alignment revealed that the SARS-CoV-2M^pro^ shares a 96% similarity to that of SARS-CoV-1. This enzyme is reported to be inhibited by several classes of compounds, some of which exhibit anti-CoV activities *in vitro*, *in vivo*, and even in nonrandomized trials (such as lopinavir).^7^ RNA-dependent RNA polymerase (RdRp) is a key enzyme essential for the viral replication of SARS-CoV-2.^13^ Due to their crucial roles, these viral proteins are considered imperative targets for developing antiviral compounds against COVID-19. The SARS-CoV-2 genome encodes more than 20 proteins, which include the main protease (M^pro^), a 3C-like protease (3CLP) that shares 96.1% similarity with 3CLP of SARS-CoV.^7^ M^pro^, a homodimeric cysteine protease, plays an important role in SARS virus replication and transcription. When the mRNA of the virus is translated polyproteins, M^pro^ is first auto-cleaved to become a mature enzyme, which in turn cleaves all of the 11 remaining downstream nonstructural proteins of the polyproteins to polypeptides, which are required for the replication process of the virus. The prime anti-COVID-19 target proposed is the main protease (M^pro^; also known as 3 chymotrypsin like protease or 3CL^pro^), which is regarded as responsible for the cleavage of viral peptides into functional units for virus replication and packaging within the host cells. Likewise, the enzyme RNA dependent RNA polymerase (RdRp) responsible for viral RNA synthesis is also considered a druggable target to inhibit SARS-CoV-2. Conceivably, the interaction of viral spike proteins with its receptor, angiotensin-converting enzyme-2 (ACE2) on host cells, and subsequent viral endocytosis into the cells, may also are regarded as a viable drug target. Similarly, the enzyme transmembrane protease serine-2 (TMPRSS2) produced by the host cells plays an important role in proteolytic processing of S protein priming to the receptor ACE2 binding in humans and has gained attention.^14–18^ Whilst, these targets have been currently investigated extensively with numerous drugs and biologicals, the poly targeting approaches such as the leads having the potential to act on synergistic pathways concurrently by which SARS-CoV-2 infection is inhibited in the host cells. The cytokines, including interleukins, play a vital role in regulating host cell immune responses. Upon the SARS-CoV infection, a rapid and severe immune reaction occurs, and this triggers the synthesis and release of relatively higher levels of interleukins into the bloodstream. Consequently, the raise of pro-inflammatory cytokines leads to severe respiratory organ failure, high fever, and other associated diseases.^19^ Owing to these reasons, we hypothesize that the drug candidates having the potential to elicit anti-viral effects on multiple targets along with beneficial immunomodulatory effects in host cells, organs or systems would enable novel therapeutic leads to combat the COVID-19 pandemic. Thus, our study intended to search for the leads having the potential to act on multi druggable targets of COVID-19 along with beneficial immunomodulatory effects.

Whilst, the drug discovery and developmental research has undergone significant modernization in recent decades, the three fourth of humankind still depend largely on the bioactive from natural sources.^20^ The plants and their parts have been serving human mankind as sources of herbal medicine since ancient times. They possess a variety of constituents, such as flavonoids, alkaloids, glucosides, and polyphenolic compounds. These phytochemicals offer a wide range of therapeutic properties and novel scaffolds to design new drugs.^21,22^ Of note, several phytochemicals have been reported beneficial in preventing or treating zoonotic viral diseases including SARS and MERS-CoV. The plant constituents having bi, mono &heterocyclic rings specially bioflavonoids adapt to the geometry of the 3CL^pro^ substrate site and have prominent interactions with the surrounding amino acid residues.^23^ This is further substantiated by the findings of cannabisin A and isoacetoside as potential 3CL^pro^ inhibitors by Free Energy Perturbation studies.^24^ In regards, we have collated the potent phytochemicals which have been previously reported to inhibit SARS and MERS-CoV. This study aims to narrow and sort the selective 132 phytomolecules (ESI Table 1†) based on its binding affinities and interactions on major druggable target of COVID-19 using *in silico* computational tools. Besides, the analogues of the sorted lead molecule were prepared and examined for its molecular docking, ADMET, and molecular dynamics.

## Materials and methods

### Docking software and ligand library

**Fig. 1:**
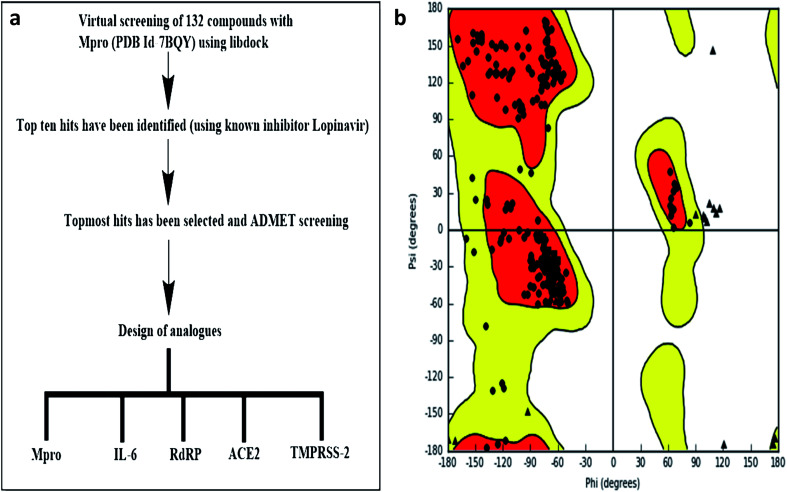
(a) Experimental workflow (b) Ramachandran plot of M^pro^.

LIBDOCK and CDOCKER modules of Discovery Studio 3.5 software (DS3.5, Accelrys, Inc., San Diego, CA, USA) were utilized for virtual screening and ligand binding analysis of inhibitors. A library consisting of 132 plants secondary metabolites already reported for SARS-CoV (2003) and MERS-CoV (2012), and other viruses after sessional outbreaks caused by dengue and influenza virus was generated (ESI Table 1†). All phytochemicals were screened for M^pro^ and the top hit and its analogs were further docked with the proteins M^pro^s, RdRp, ACE-2, TMPRSS2, and IL-6. The binding energies and the interactions were compared with the known inhibitors of the selected proteins Lopinavir, Favipiravir, Ramipril, camostat, and tartaric acid respectively. A detailed workflow is depicted in Fig. 1a.

### Structure-based virtual screening using LIBDOCK

LIBDOCK is a rigid-based docking program. It calculates hotspots for the protein using a grid placed into the binding site and using polar and polar probes (San Diego, CA, USA). Then the hotspots are further used to align the ligands to form favorable interaction. After minimized, all the ligand poses are ranked based on the ligands score. The 1.7 Å crystal structure of CoV main proteases (M^pro^s) in complex with N3 a mechanism-based inhibitor (PDB ID: 7BQY) were downloaded from the protein data bank (PDB) and imported to the working environment of LIBDOCK. The protein was prepared by removing crystal water and other heteroatoms (except N3), followed by the addition of hydrogen, protonation, ionization, and energy minimization. The CHARMm (Cambridge, MA, USA)^25^ force field and the Smart minimizer algorithm were applied for energy minimization. The minimization performed 2000 steps with an RMS gradient tolerance of 0.1, and the final RMS gradient was 0.09463. The prepared protein was used to define the binding site from the “Edit binding site” option on the receptor–ligand interaction toolbar. Using the bound ligands (N3) binding positions, the active sites for docking were generated. Virtual screening was carried out by docking all the prepared ligands at the defined active site using LIBDOCK. Based on the LIBDOCK score, all the docked poses were ranked and grouped by name. All compounds were ranked according to their LIBDOCK score.

The molecular docking (CDOCKER) module of Discovery Studio was used for the molecular docking study. CDOCKER is an implementation of a CHARMm based docking tool. The receptor is held rigid while the ligands were allowed to flex during the docking process. For each complex pose, the CHARMm energy (interaction energy plus ligand strain) and the interaction energy, which indicate ligand binding affinity was calculated. The crystal structures of M^pro^s (PDB ID: 7BQY, 1.7 Å), RdRp (PDB ID: 6WTC, 1.85 Å), ACE-2 (PDB ID: 6ACK, 4.5 Å), TMPRSS-2 (PDB ID: 2E7V, 1.92 Å) and IL-6 (PDB ID: 1ALU, 1.90 Å)^26^ were obtained from the protein data bank. The crystal water molecules were generally removed in rigid and semi-flexible docking process^27,28^ since the fixed water molecules might affect the formation of the receptor–ligand complex. The water molecules were removed, and hydrogen atoms were added to the protein. The structure was completed by modelling the missing loops. The missing atoms were inserted by standardizing the names of the atom. The titratable residues were protonated by p*K*_a_ prediction. The potential energy van der Waals energy, Electrostatic energy, and RMS gradient of the complexes were checked before and after protein minimization. Finally, the hydrogen receptors were merged to the target receptor molecule using discovery studio 4.1 clients.^29^ The prepared proteins were validated through a Ramachandran plot (Fig. 1b).^25^ The selected plant constituents were generated and optimized using ChemSketch software. All the tautomer's and isomers of the ligand were regarded and of the ligands possessing the lowest energy had been approved for docking.

The CHARMm force field was used for energy minimisation. The binding site spheres of all the proteins were defined as the regions that come within a radius of 15 Å from the geometric centroid of the native ligands. The binding site spheres of all the proteins were defined by structural alignment. During the docking process, the ligands were allowed to bind to the residues within the binding site spheres. After being extracted from the binding site, the native ligands were re-docked into the crystal structure of all proteins. The RMSD between the docked pose and the crystal structure of the complex was less than 1 Å, indicating the CDOCKER module was highly reliable for reproducing the experimentally observed binding mode of all proteins. The structures of identified hits were prepared and docked into respective proteins. Different poses for each test molecule were generated and analyzed based on CDOCKER interaction energy. The designed compounds were minimized and used as input ligands in the protocol explorer of CDOCKER. The molecular dynamic protocol was used to generate various conformations for ligand and the initially generated structures were refined using a simulated annealing protocol. CDOCKER energies were calculated for each compound. The various binding interactions between the ligand and protein were predicted. The protocol uses a CHARMm-based molecular dynamics (MD) scheme to dock ligands into a receptor binding site. Random ligand conformations are generated using high-temperature MD. The conformations are then translated into the binding site. Candidate poses are then created using random rigid-body rotations followed by simulated annealing. A final minimization is then used to refine the ligand poses. The CDOCKER energy as an estimation of molecular complex binding affinity was used in this study.

### Molecular dynamics

The molecular dynamic (MD) simulation was conducted to study the stability of the ligand–target complex.^30^ The simulation is set to constant number (*N*), volume (*V*), and temperature (*T*) (*NVT*) ensemble with temperature varying from 300 K, run: 80 ns, time step: 0.002, and quality threshold (QT) (the temperature response set enforce constant temperature in a molecular dynamics simulation) is set to 0.2 (Fig. 1c). It took 12.55 h to complete the simulation. The ligand–target complexes were then subjected to molecular dynamics simulation using NAMD with the following parameters; minimization-2000 steps; annealing-144 000 steps; equilibration & MD-500 000 steps; *NVT* temperature 27 °C/300 K; NPT pressure-1 atm and simulation time-80 ns.

The highly constructive conformation, pose and favorable bonded complexes form receptor ligand interaction was used an input to study molecular dynamics simulation (MD) for the compound GC-6. The CHARMm (Chemistry at Harvard Macromolecular Mechanics) force field were applied to each complex system followed by two step minimization of 500 cycles with steep descent (SD) and conjugate gradient (CG) to eliminate possible steric strain. The relaxed and energy minimized complex were then subjected to three steps of dynamics and simulation. Initially, receptor–ligand complex was gradually simulated and heated from a temperature of 50 K followed by its ramping to 300 K and then equilibrated by 100 steps at 310 K for even distribution of atoms in a system (protein–ligand) to be constrained. Finally, production of CHARMm simulation was performed for 80 ns and conformations were allowed to save for every 2 ps. Furthermore, the Spherical Cutoff method of electrostatics was implemented to study the all-non-bonded energy. In order to fix hydrogen bonding during simulation leap frog verlet a dynamics integrator was introduced with SHAKE constraint. Equilibration of the complex system was monitored by convergence in terms of the H-bond, root-mean-square fluctuation (RMSF) and the RMSD (root-mean-squared deviations).

## Results

### Virtual screening of phytochemicals database against CoV main proteases (M^pro^s)

The prime target, coronavirus main protease (M^pro^s), which is a chief culprit enzyme, guides in mediating the transcription and replication process of the virus^31^ has been assigned for our study. There are ∼30 000 nucleotides have compressed together in the virus genome of novel COVID-19, its gene may perhaps encode with two polyproteins namely, pp1a and pp1ab, which are desirable for viral replication and transcription.^32,33^ The main Proteolytic enzyme, M^pro^s, through the Proteolytic process could release the polypeptides from these polyproteins. Besides, this M^pro^ enzyme is the main protein in the viral cell life cycle together with the absence of closely related homolog's of human protein, recognizes the M^pro^s as a promising target for the anti-viral drug discovery. Therefore, the inhibition of M^pro^ may perhaps block the biosynthesis of the viral polyprotein, resulting in viral death. To identify new compounds that could potentially inhibit M^pro^s through binding to the ligand binding pocket, virtual screening was carried out using the LibDock module of DS.

The top 10 phytochemicals which have higher binding affinities with M^pro^ relative to all docked molecules are listed in Table 1. The binding affinities obtained for phytochemicals were compared with a conventional M^pro^ inhibitor, Remdesivir which is also extensively used for the treatment of COVID-19. The results reveal that the phytochemicals namely isotheaflavin 3′-gallate, dieckol, gallocatechin gallate, broussoflavan A, and hygromycin B have shown LibDock scores similar to Remdesivir (LibDock score: 177.63). Of note, the LibDock score of cinnamtannin B1 was higher than the score obtained for Remdesivir.

**Table 1:** Top 10 ranked compounds with likely LibDock scores of Remdesivir

S. no.	Molecule	LIBDOCK Score
1	Isotheaflavin 3′-gallate	179.72
2	Cinnamtannin B1	184.88
3	Dieckol	175.51
4	Gallocatechin gallate	173.33
5	Broussoflavan A	172.76
6	Hygromycin B	172.23
7	Epi-gallocatechin gallate	167.03
8	Ginkgetin	165.38
9	Amentoflavone	165.32
10	Epi-catechin gallate	164.68
11	Remdesivir	177.63

### Ligand binding analysis

**Fig. 2:**
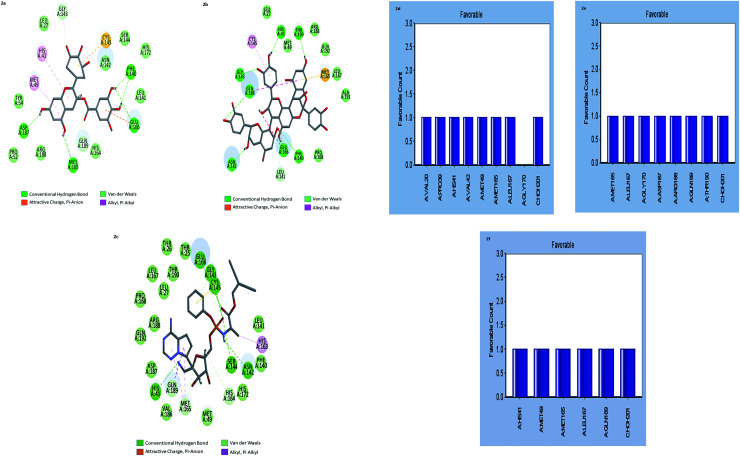
(a–c) the inter-molecular interaction of the predicted binding modes of (a) gallocatechin gallate; (b) cinnamtannin B1 and (c) Remdesivir to M^pro^s. (d–f) Bar diagrams depicting the favourable interactions of the residues of M^pro^s towards (d) gallocatechin gallate (e) cinnamtannin B1 and (f) Remdesivir.

To study ligand binding mechanisms, the top six ranked compounds isotheaflavin 3′-gallate, dieckol, gallocatechin gallate, broussoflavan A, hygromycin B, and cinnamtannin B1 were docked into the 3D structure of (M^pro^s) by using the CDOCKER module of DS. The CDOCKER energy as an estimation of molecular complex binding affinity was used in this study. As shown in Table 2, the CDOCKER energy of gallocatechin gallate and cinnamtannin B1 were lower than the reference Remdesivir, indicating that these two compounds elicit a higher binding affinity with M^pro^s. The energy of the other compound dieckol is close to Remdesivir. However, the interaction energy of compounds broussoflavan, isotheaflavin 3′-gallate, and hygromycin were not significant when compared to the reference Remdesivir. The increased binding energies of gallocatechin gallate and cinnamtannin B1 were further supported by the comparative analysis of receptor–ligand interactions with the active amino acid residues of M^pro^s. The major non-bonded interactions such as hydrophilic, hydrophobic, and pi–pi interactions of these two compounds were investigated and compared with the standard Remdesivir (Fig. 2a–c ESI Tables 2 and 3†).

**Table 2:** CDOCKER energies of selected phyto-constituents

S. no.	Molecule	-CDOCKER interaction energy (kcal mol^−1^)	Pose
1	Gallocatechin gallate	43.69	1
2	Cinnamtannin B1	34.54	1
3	Dieckol	29.90	1
4	Broussoflavan A	4.85	1
5	Isotheaflavin 3′-gallate	22.85	1
6	Hygromycin B	−9.57	1
7	Remdesivir	33.84	1

The results showed that both gallocatechin gallate and cinnamtannin B1 formed eight and seven pairs of hydrogen bonds with M^pro^s, respectively whereas the drug Remdesivir has shown five pairs of hydrogen bonds only. The additional hydrogen bonds formed may be the reason for the increased binding affinity of these two compounds than Remdesivir. Both of the compounds formed three pair Pi–Pi interactions with M^pro^s, by the centroid of the benzene ring of methionine and the centroid of the benzene ring of gallocatechin gallate and cinnamtannin respectively. The interactions of the amino acid residues are depicted in (Fig. 2d–f). These results indicated that gallocatechin gallate and cinnamtannin B1 probably bind to M^pro^s with similar or even better affinity than Remdesivir.

Design of gallocatechin analogs Lipinski rule of five (L-ROF) is an important parameter for oral drug candidates. When we analyzed the L-ROF of two compounds gallocatechin gallate and cinnamtannin, violations of the gallocatechin were 3 and 2 respectively (Table 3).

**Table 3:** Lipinski rule prediction of gallocatechin (GA) and cinnamtannin B (CT)

Molecule	log *P*	Mol. wt. (g)	H bond donor	H bond acceptor	No. of violations
GA	0.25	460.4	8	11	2
CT	3.3	864.8	14	18	3

It is a well-known fact that beyond the violation of 2 is questionable. Based on these results, we decided to proceed with gallocatechin further. Chemical modification of naturally derived compounds may be required to increase the potency.

**Fig. 3:**
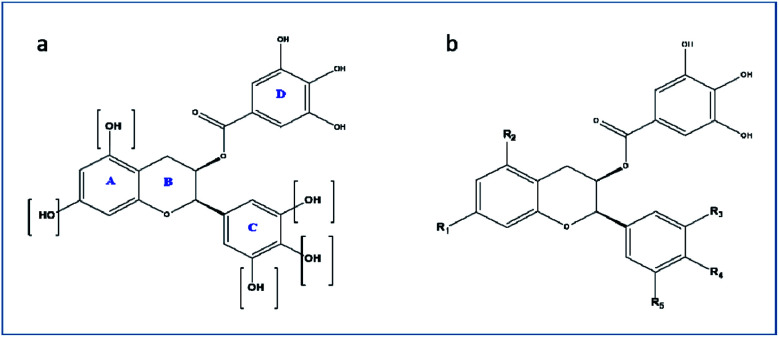
(a and b) Gallocatechin gallate scaffold.

For the drug discovery process, beginning with the understanding of the structural conformity (*i.e.* structure including potential isomers) of the naturally derived compound(s) can timelines and greatly increase the chance. Working on this principle we designed six analogs of gallocatechin (Fig. 3a and b). To alter the hydrogen bond donor/acceptor and to increase the hydrophobicity, the OH groups of ring A and C of gallocatechin are removed in a sequential manner to derive the analogs (Table 4).

**Table 4:** Substitution pattern of designed gallocatechin analogues of finding effective viral inhibitors

Compound	R_1_	R_2_	R_3_	R_4_	R_5_
GC-1	OH	OH	OH	OH	OH
GC-2	OH	OH	H	H	H
GC-3	OH	H	OH	OH	OH
GC-4	OH	H	H	H	H
GC-5	OH	H	OH	H	H
GC-6	H	H	OH	OH	OH
GC-7	H	H	H	H	H

The designed analogs were further docked with the proteins M^pro^s, RdRp, ACE-2, TMPRSS2, and IL-6. The binding energies and the interactions were compared with the known inhibitors of the selected proteins lopinavir, favipiravir, Ramipril, camostat, and tartaric acid respectively. As shown in Table 5 the CDOCKER energies of designed analogs were compared with known inhibitors. The selected compounds have shown the least CDOCKER energy towards ACE-2, IL-6, and RdRp among the five targets compared to the known standards (>2.11781, 19.1244, and 3263 kcal mol^−1^) respectively. Against COVID main protease, and transmembrane protease serine-2 proteins, the CDOCKER energy of tested compounds were not significant. The energies were bigger than the standard lopinavir (<51.2735 kcal mol^−1^), (<48.1854). However, the compounds GC-3 and GC-6 have shown the CDOCKER energies which are nearing the standard. Interestingly, the CDOCKER energies of tested compounds were lower towards the proteins ACE-2, IL-6, and RdRp than the reference compounds Ramipril, tartaric acid, and favipiravir, respectively. These results indicating the compounds may possess COVID-19 combating activity through the inhibition of these proteins. Among all, compound GC-3 and compound GC-6 shows significant activity towards all the enzymes. However, a further detailed *in vitro* study is needed to prove this concept.

**Table 5:** CDOCKER interaction energies of designed compounds

Compound	-CDOCKER interaction energy (kcal mol^−1^)				
	M^PRO^	TMPRSS2	ACE-2	IL-6	RdRP
GC-1	41.61	36.07	30.40	31.69	42.11
GC-2	34.12	31.73	22.23	24.78	30.58
C-3	44.07	37.68	28.49	31.64	37.37
GC-4	35.24	32.41	22.25	28.53	33.80
GC-5	37.82	33.60	21.98	26.43	33.28
GC-6	40.60	37.83	25.43	31.54	43.09
GC-7	33.53	27.87	17.13	23.34	27.96
Lopinavir	49.42	—	—	—	—
Ramipril	—	—	25.12	—	—
Camostat	—	54.24	—	—	—
Favipravir	—	—	—	—	22.57
Tartaric acid (co-crystal)	—	—	—	17.61	—

### Molecular dynamics study

**Fig. 4:**
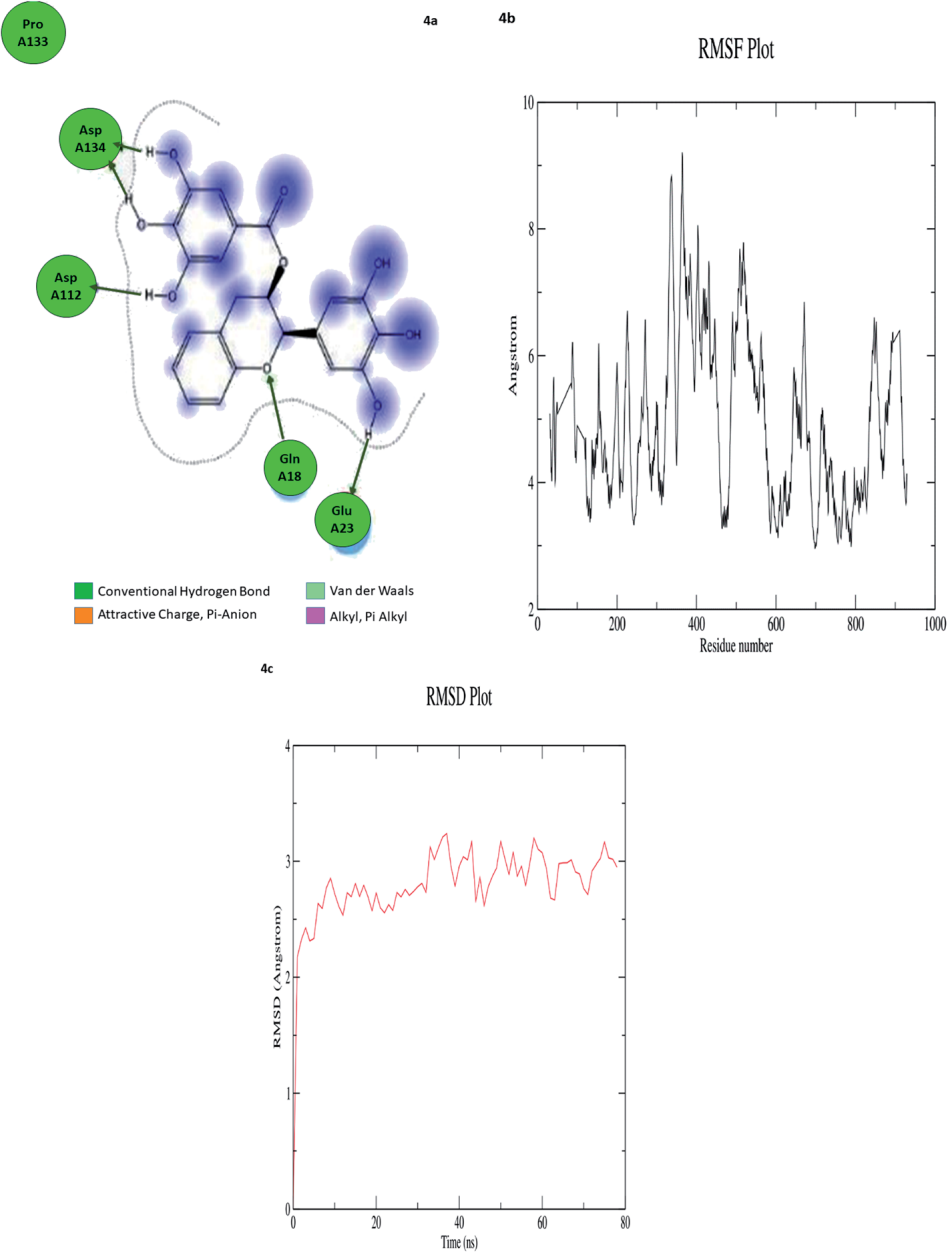
(a) Hydrogen bond interactions (b) RMSD graph (c) RMSF graph of GC-6 and 6WTC complex.

In computational drug discovery, MD is a technique that sheds light on the allosteric binding site of the protein, and conformation of the ligand–protein complex. Also, it can simulate the conditions that are hard to perform in wet experiments. Herein, compound GC-6 was subjected to MD simulations bound to the SARS-CoV-2 RdRp. The initial conformations were acquired from the molecular docking experiments by CDOCKER. It can be seen from Fig. 4a that compound GC-6 retained its binding affinity and was firmly bound to its respective binding site. The RMSD curves of the receptor structures from each complex and the potential energy profiles of each complex are shown in (Fig. 4b and c).

The trajectories of complexes reached equilibrium after 10 ns, RMSD, and the potential energy of the complexes gets stabilized with time. The H-bond distances formed between GC-6 and RdRp were within a range of around 3.0 and 2.3 Å. Besides, GC-6 formed three pairs of hydrogen bonds with the water molecules after molecular dynamics simulation (Table 6). These hydrogen bonds might contribute to the stability of the complexes. The RMSD values of each receptor-ligand complex were plotted to elucidate the effect of the compounds on the structural stability and integrity. From this plot, 6WTC_GC-6 showed sharp increase in RMSD up to 2 nano seconds. Later 6WTC_GC-6 deviations are optimized up to 80 nano seconds, then it increase slightly to 0.75 Å further the complex attained equilibrium till the end of dynamics.

**Table 6:** The ligand target interaction after MD simulation

Ligand	Interaction score (kcal mol^−1^)	Ligand atoms (number)	Receptor atoms (number)	Residue (number)	Chain (number)	Type	Score (%)	Distance (Å)
GC-6	−9.2003	H 5948	OE 3340	GLU 23	6WTC 3	H-donor	21.60	1.42
		H 5962	O 1702	ASP 112	6WTC 2	H-donor	25.00	3.64
		H 5964	O 1702	ASP 112	6WTC 2	H-donor	38.20	1.77
		H 5962	OD 2043	ASP 134	6WTC 2	H-donor	42.40	1.23
		H 5966	OD 2043	ASP 134	6WTC 2	H-donor	34.00	1.53

Considering all the evaluation indexes, the compound GC-6 might interact with RdRp steadily and have potential negative modulator effects on RdRp.

## Discussion

One family of compounds that demonstrate antiviral activity across several studies is the polyphenols. It was well known that hydrophobicity plays a major role in predicting the binding affinity towards SARS-CoV-1. Several natural sources from flora and fauna have been tested in terms of their anti-SARS-CoV-1 activity and used as a scaffold in drug development since its outbreak in 2003 (ESI Table 1). These natural metabolites include flavones, flavonols, fatty acids, tannins, terpenes, and alkaloids.

The diversity of these chemical classes is related to the different mechanisms used by each phytochemicals class capable to inhibit coronaviruses. Jo *et al.* (2019) stated that SARS-CoV-1 inhibition requires chemical structures containing a hydrophobic aromatic ring, hydroxyl groups, and carbohydrates moieties. Although not all of the anti-SARS-CoV-1 compounds (ESI Table 1†) present the aromatic ring, their molecular structures have lipophilic and hydrophilic regions and the ability to form multiple hydrogen bonds through hydroxyl groups. It was well evidenced by our results that the identified compound has hydrophobic and multiple hydroxyl groups. Several investigations revealed the anti-SARS-CoV-2 activity of catechins or polyphenols through the inhibitory potential of M^Pro^S alone, which have been highlighted by R. Ghosh *et al.* recently. Although the importance of M^Pro^S has been established as a prime target for COVID-19, there are targets like RdRp and ACE-2. TMPRSS-2 and IL-6 have not been explored yet. There were also limited studies of the polyphenols and catechins against the mentioned targets. Poly targeting is also a reasonable approach to avoid issues related to resistance and toxicity.

Keeping all these views, we wanted to study the GC and its analogs against these multiple targets. Interestingly, we observed the best binding affinities of GC against the targets RdRp, ACE-2, TMPRSS2, and IL. Though it is a potential HIT against these multiple targets, it fails to satisfy L-ROF. L-ROF is a very basic filter for oral drug candidates. In view of these, we designed analogs of GC where the OH groups have been modified (to satisfy HBD = 5; HBA = 10) and all were within the range (0–1 violation) (Table 5). All the designed analogs (GC-2 to GC-7) have shown a comparable binding affinity with GC itself; however, the compound GC-6 was identified as the topmost HIT. By considering binding energies, amino acid residues interactions nod molecular dynamics studies, we propose the following pharmacophoric query for having multitarget inhibitory potential of SARS-CoV-2. Ring aromatics >3; hydrogen bond acceptors >8; hydrogen bond donors >5.

## Conclusion

The rapidly spreading outbreak of COVID-19 has challenged the healthcare sector of the world in the last few months. Several investigations have demonstrated that a variety of plant secondary metabolites especially polyphenolic have been reported for anti-SARS-CoV-1& 2 activities since 2002. However, there is very little evidence as yet that these constituents work to prevent or treat SARS-CoV-1& 2 in humans. Moreover, these plant secondary metabolites inhibit anyone's target and not much of their analogs have been reported so far against this SARS CoV-2. To overcome the less efficacious/resistance problems of plant secondary metabolites against SARS CoV-2, we aimed to identify any single molecule that can target all important targets involved in SARS CoV-2. A systematic structure based *in silico* study was carried out to meet our objective. A gallocatechin analog namely (2*R*,3*R*)-2-(3,4-dihydroxyphenyl)chroman-3-yl-3,4,5-trihydroxy benzoate has been identified as a multiple targeting inhibitor. Gallocatechin gallate, a catechin and a constituent of green tea, is an epimer of epigallocatechin gallate. There were strong evidences at *in silico* level for its effect SARS CoV-2 therapy. An *in silico* molecular docking study with SARS CoV-2 3CL protease showed that EGCG strongly interacted with M^pro^ indicating that EGCG potentially inhibit SARS CoV-2. 3CL protease-cleavage of viral proteins by protease is a vital step for viral replication.^34–36^

Further, it is supported by Minsu Jang *et al.*, that EGCG inhibits 3CL protease activity of SARS-CoV-2 in dose dependent manner and the half inhibitory concentration (IC_50_) was 7.58 μg ml^−1^ examine the effect of EGCG on coronavirus. EGCG treatment decreases 3CL-protease activity of HCoV-OC43 and HCoV-229E. Moreover, EGCG treatment decreased HCoV-OC43-induced cytotoxicity. Finally, they also found that EGCG treatment decreased the levels of coronavirus RNA and protein in infected cell media. These results indicate that EGCG inhibits coronavirus replication.^37,38^ In another study, the polyphenol EGCG exhibited significant effect on various druggable targets of SARS CoV-2. These results suggest the potential activity of tea polyphenols in the treatment of COVID-19. The use of tea phyto constituents over synthetic drugs is an exciting treatment option since they are safer, and a higher dose is feasible. However, the bulkiness of these polyphenols and violations of Lipinski's rules could be hindrances in their development. Oral bioavailability is a concern for these polyphenols for which to overcome their derivatives are being developed. EGCG derivatives are being tested for enhancement in physicochemical properties and better efficacy. In line of these, we wanted to explore the efficacy of gallocatechin gallate against various druggable targets of SARS-CoV-2.^39^ However, further studies are warranted for the validation of these compounds using *in vitro* and *in vivo* models to pave a way for these compounds in anti-COVID-19 drug discovery.

## Conflicts of interest

No potential conflicts of interest are reported by the authors. There are no conflicts to declare.

## Supplementary Material

RA-011-D1RA01603B-s001

## References

[cit1] Zhu N., Zhang D., Wang W., Li X., Yang B., Song J., Zhao X., Huang B., Shi W., Lu R., Niu P., Zhan F., Ma X., Wang D., Xu W., Wu G., Gao G. F., Tan W. (2020). N. Engl. J. Med..

[cit2] Li Q., Guan X., Wu P., Wang X., Zhou L., Tong Y., Ren R., Leung K. S. M., Lau E. H. Y., Wong J. Y., Xing X., Xiang N., Wu Y., Li C., Chen Q., Li D., Liu T., Zhao J., Liu M., Tu W., Chen C., Jin L., Yang R., Wang Q., Zhou S., Wang R., Liu H., Luo Y., Liu Y., Shao G., Li H., Tao Z., Yang Y., Deng Z., Liu B., Ma Z., Zhang Y., Shi G., Lam T. T. Y., Wu J. T., Gao G. F., Cowling B. J., Yang B., Leung G. M., Feng Z. (2020). N. Engl. J. Med..

[cit3] Cheng S.-C., Chang Y.-C., Fan Chiang Y.-L., Chien Y.-C., Cheng M., Yang C.-H., Huang C.-H., Hsu Y.-N. (2020). J. Formosan Med. Assoc..

[cit4] Zhou P., Yang X.-L., Wang X.-G., Hu B., Zhang L., Zhang W., Si H.-R., Zhu Y., Li B., Huang C.-L., Chen H.-D., Chen J., Luo Y., Guo H., Jiang R.-D., Liu M.-Q., Chen Y., Shen X.-R., Wang X., Zheng X.-S., Zhao K., Chen Q.-J., Deng F., Liu L.-L., Yan B., Zhan F.-X., Wang Y.-Y., Xiao G.-F., Shi Z.-L. (2020). Nature.

[cit5] Masters P. S. and Perlman S., Fields virology, 2013, vol. 1, pp. 825–858

[cit6] Cui J., Li F., Shi Z.-L. (2019). Nat. Rev. Microbiol..

[cit7] Ferraz W. R., Gomes R. A., Novaes A. L. S. (2020). Future Med. Chem..

[cit8] Ghosh A. K., Brindisi M., Shahabi D., Chapman M. E., Mesecar A. D. (2020). ChemMedChem.

[cit9] Hagar M., Ahmed H. A., Aljohani G., Alhaddad O. A. (2020). Int. J. Mol. Sci..

[cit10] Shahinshavali S., Hossain K. A., Kumar A. V. D. N., Reddy A. G., Kolli D., Nakhi A., Rao M. V. B., Pal M. (2020). Tetrahedron Lett..

[cit11] Zhang L., Lin D., Sun X., Curth U., Drosten C., Sauerhering L., Becker S., Rox K., Hilgenfeld R. (2020). Science.

[cit12] Gentile D., Patamia V., Scala A., Sciortino M. T., Piperno A., Rescifina A. (2020). Mar. Drugs.

[cit13] Ravi A. V. (2020). Front. Microbiol..

[cit14] Sheahan T. P., Sims A. C., Leist S. R., Schäfer A., Won J., Brown A. J., Montgomery S. A., Hogg A., Babusis D., Clarke M. O., Spahn J. E., Bauer L., Sellers S., Porter D., Feng J. Y., Cihlar T., Jordan R., Denison M. R., Baric R. S. (2020). Nat. Commun..

[cit15] Kadam R. U., Wilson I. A. (2017). Proc. Natl. Acad. Sci. U. S. A..

[cit16] Hoffmann M., Kleine-Weber H., Schroeder S., Krüger N., Herrler T., Erichsen S., Schiergens T. S., Herrler G., Wu N.-H., Nitsche A., Müller M. A., Drosten C., Pöhlmann S. (2020). Cell.

[cit17] Li G., De Clercq E. (2020). Nat. Rev. Drug Discovery.

[cit18] Li Y., Xie Z., Lin W., Cai W., Wen C., Guan Y., Mo X., Wang J., Wang Y., Peng P., Chen X., Hong W., Xiao G., Liu J., Zhang L., Hu F., Li F., Zhang F., Deng X., Li L. (2020). Med.

[cit19] Mesaik M. A., Jabeen A., Halim A., Begum A., Shukralla A., Asif M., Ul-Haq Z., Iqbal M. (2012). Chem. Biol. Drug Des..

[cit20] Cragg G. M., Newman J. D. J. (2013). Biochim. Biophys. Acta.

[cit21] Aanouz I., Belhassan A., El-Khatabi K., Lakhlifi T., El-ldrissi M., Bouachrine M. (2020). J. Biomol. Struct. Dyn..

[cit22] Gupta M. K., Vemula S., Donde R., Gouda G., Behera L., Vadde R. (2020). J. Biomol. Struct. Dyn..

[cit23] Olubiyi O. O., Olagunju M., Keutmann M., Loschwitz J., Strodel B. (2020). Molecules.

[cit24] Ngo S. T., Quynh Anh Pham N., Thi Le L., Pham D.-H., Vu V. V. (2020). J. Chem. Inf. Model..

[cit25] Brooks B. R., Brooks C. L., Mackerell A. D., Nilsson L., Petrella R. J., Roux B., Won Y., Archontis G., Bartels C., Boresch S., Caflisch A., Caves L., Cui Q., Dinner A. R., Feig M., Fischer S., Gao J., Hodoscek M., Im W., Kuczera K., Lazaridis T., Ma J., Ovchinnikov V., Paci E., Pastor R. W., Post C. B., Pu J. Z., Schaefer M., Tidor B., Venable R. M., Woodcock H. L., Wu X., Yang W., York D. M., Karplus M. (2009). J. Comput. Chem..

[cit26] Jin Z., Du X., Xu Y., Deng Y., Liu M., Zhao Y., Zhang B., Li X., Zhang L., Peng C., Duan Y., Yu J., Wang L., Yang K., Liu F., Jiang R., Yang X., You T., Liu X., Yang X., Bai F., Liu H., Liu X., Guddat L. W., Xu W., Xiao G., Qin C., Shi Z., Jiang H., Rao Z., Yang H. (2020). Nature.

[cit27] Beard H., Cholleti A., Pearlman D., Sherman W., Loving K. A. (2013). PLoS One.

[cit28] Sarvagalla S., Singh V. K., Ke Y.-Y., Shiao H.-Y., Lin W.-H., Hsieh H.-P., Hsu J. T. A., Coumar M. S. (2015). J. Comput.-Aided Mol. Des..

[cit29] Nganou B. K., Tane P., Nchiozem A., Jubie Selvaraj S. A., Nanjan C. (2017). J. Pharm. Sci. Res..

[cit30] Yang H., Yang M., Ding Y., Liu Y., Lou Z., Zhou Z., Sun L., Mo L., Ye S., Pang H., Gao G. F., Anand K., Bartlam M., Hilgenfeld R., Rao Z. (2003). Proc. Natl. Acad. Sci. U. S. A..

[cit31] Wu F., Zhao S., Yu B., Chen Y.-M., Wang W., Song Z.-G., Hu Y., Tao Z.-W., Tian J.-H., Pei Y.-Y., Yuan M.-L., Zhang Y.-L., Dai F.-H., Liu Y., Wang Q.-M., Zheng J.-J., Xu L., Holmes E. C., Zhang Y.-Z. (2020). Nature.

[cit32] Pillaiyar T., Manickam M., Namasivayam V., Hayashi Y., Jung S.-H. (2016). J. Med. Chem..

[cit33] Ghosh R., Chakraborty A., Biswas A., Chowdhuri S. (2020). J. Biomol. Struct. Dyn..

[cit34] Ghosh R., Chakraborty A., Biswas A., Chowdhuri S. (2020). J. Biomol. Struct. Dyn..

[cit35] Peele K. A., Potla Durthi C., Srihansa T., Krupanidhi S., Ayyagari V. S., Babu D. J., Indira M., Reddy A. R., Venkateswarulu T. C. (2020). Informatics in Medicine Unlocked.

[cit36] Bhardwaj V. K., Singh R., Sharma J., Rajendran V., Purohit R., Kumar S. (2020). J. Biomol. Struct. Dyn..

[cit37] Jang M., Park R., Park Y.-I., Cha Y.-E., Yamamoto A., Lee J. I., Park J. (2021). Biochem. Biophys. Res. Commun..

[cit38] Jang M., Park Y.-I., Cha Y.-E., Park R., Namkoong S., Lee J. I., Park J. (2020). J. Evidence-Based Complementary Altern. Med..

[cit39] Mhatre S., Naik S., Patravale V. (2021). Comput. Biol. Med..

