# Supplementary material for: Identification of (2R,3R)-2-(3,4-dihydroxyphenyl)chroman-3-yl-3,4,5-trihydroxy benzoate as multiple inhibitors of SARS-CoV-2 targets; a systematic molecular modelling approach
Source: RSC Adv. 2021 Apr 7;11(22):13051–60. doi: 10.1039/d1ra01603b (PMC8697517; doi:10.1039/d1ra01603b)
Supplement: RA-011-D1RA01603B-s001 [file RA-011-D1RA01603B-s001.pdf]

Electronic Supplementary Material (ESI) for RSC Advances.
This journal is © The Royal Society of Chemistry 2021

Supplementary Data

Suppl. Table 1. Reported phytochemicals to combat SARS-CoV.

S.No	Name of phytochemical	Chemical structure
1.	Lycorine from *Lycoris radiata*	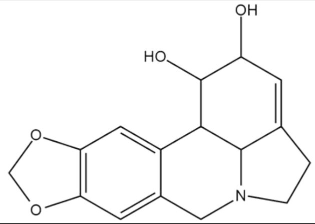
2.	Aescin	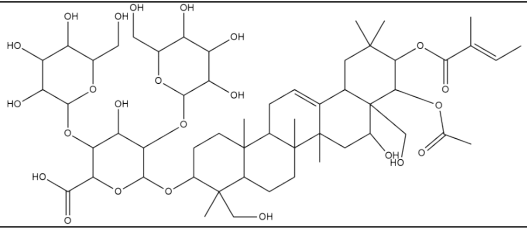
3.	Indigo from *Isatis indigotica*	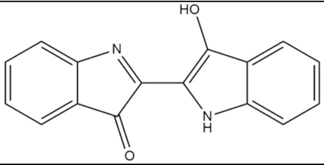
4.	Indirubin from *Isatis indigotica*	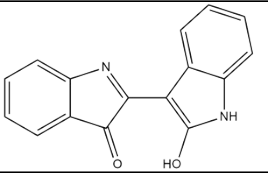
5.	Indican from *Isatis indigotica*	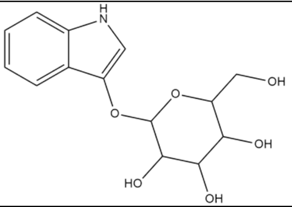
6.	Sinigrin from *Isatis indigotica*	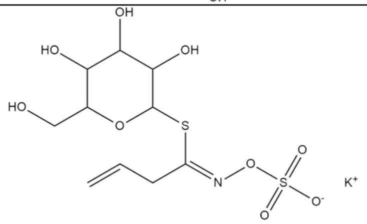
7.	β-Sitosterol from *Isatis indigotica*	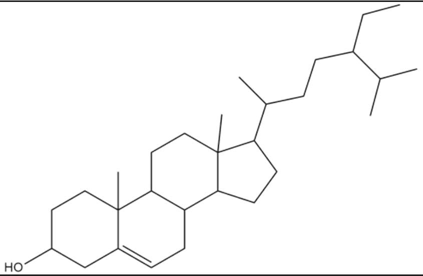

8.	Aloe emodin from *Isatis indigotica*	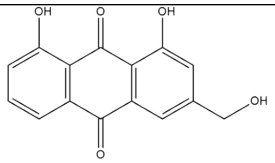
9.	Hesperetin from *Isatis indigotica*	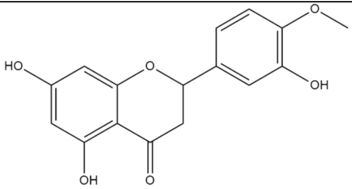
10.	18-Hydroxyferruginol from *Torreya nucifera*	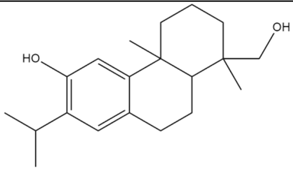
11.	Apigenin from *Torreya nucifera*	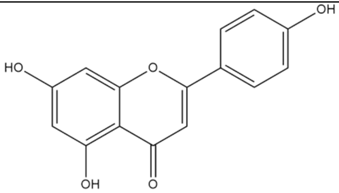
12.	Hinokiol from *Torreya nucifera*	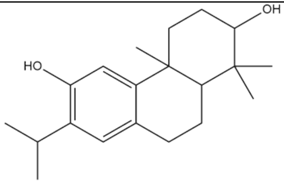
13.	Ferruginol from *Torreya nucifera*	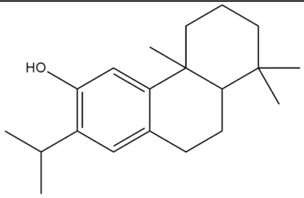
14.	18-Oxoferruginol from *Torreya nucifera*	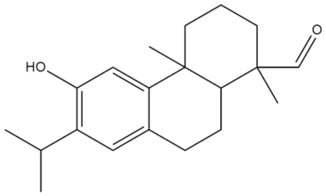
15.	*O*-Acetyl-18-hydroxyferruginol from *Torreya nucifera*	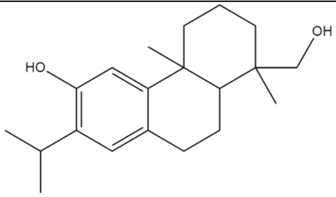

16.	Methyl dehydroabietate from *Torreya nucifera*	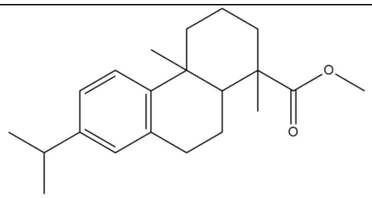
17.	Isopimaric acid from *Torreya nucifera*	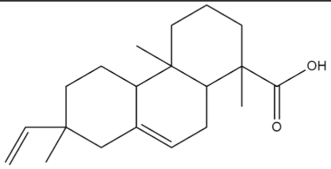
18.	Kayadiol from *Torreya nucifera*	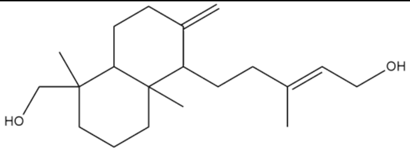
19.	Amentoflavone from *Torreya nucifera*	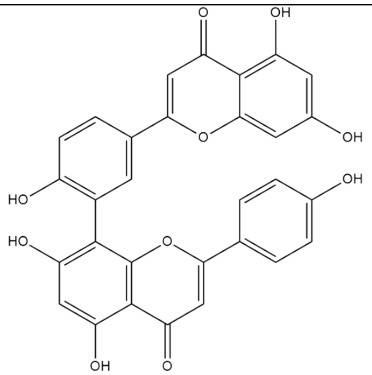
20.	Bilobetin from *Torreya nucifera*	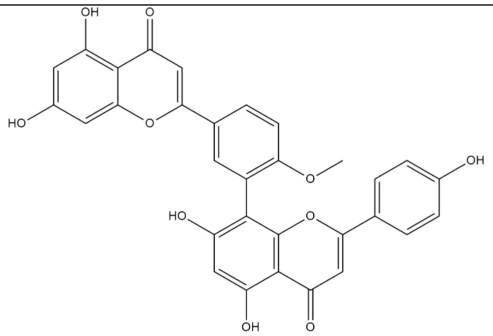
21.	Ginkgetin from *Torreya nucifera*	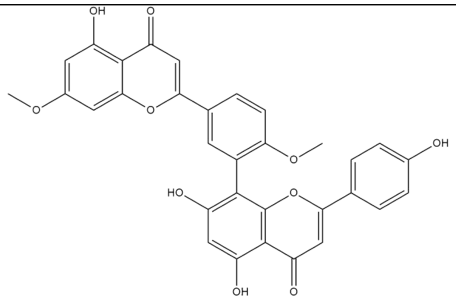

22.	Sciadopitysin from *Torreya nucifera*	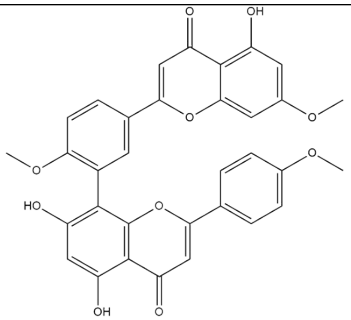
23.	Luteolin from *Torreya nucifera*	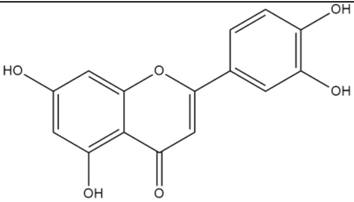
24.	Myricetin from Chromadex sp.	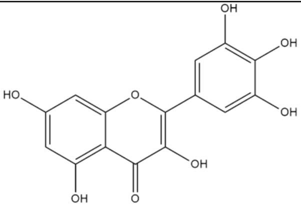
25.	Scutellarein from *Scutellaria baicalensis*	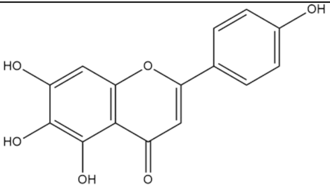
26.	Cinanserin from *Houttuynia cordata*	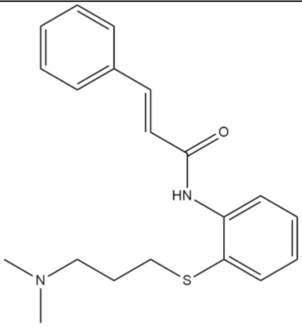

27.	Rutin from *Houttuynia cordata*	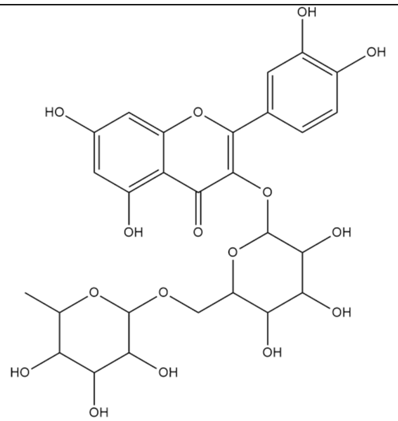
28.	Glycyrrhizin from *Glycyrrhiza glabra*	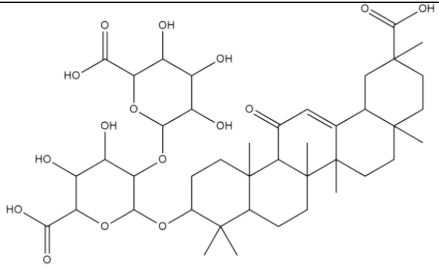
29.	Reserpine from Rawolfia *sp.*	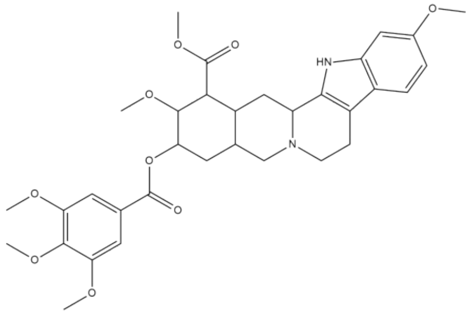
30.	Herbacetin	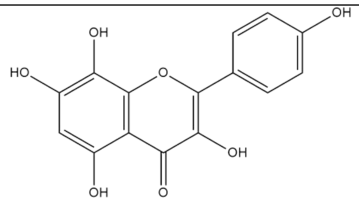
31.	Isobavachalcone	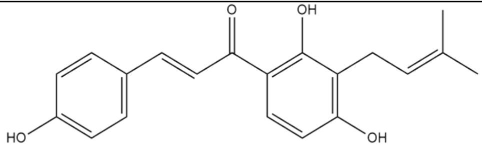
32.	Helichrysetin	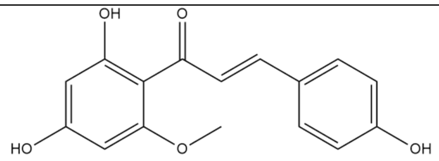

33.	Tetrandrine from *Stephania tetrandra*	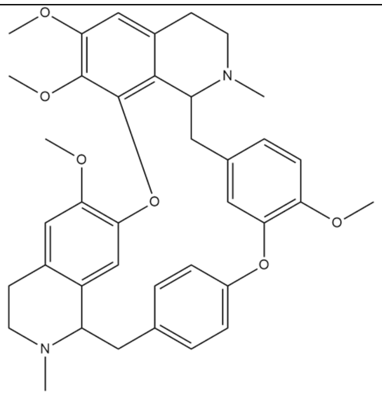
34.	Fangchinoline from *Stephania tetrandra*	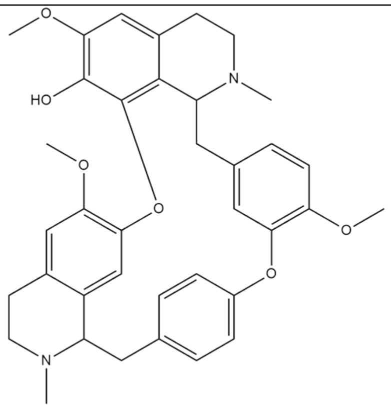
35.	Cepharanthine from *Stephania tetrandra*	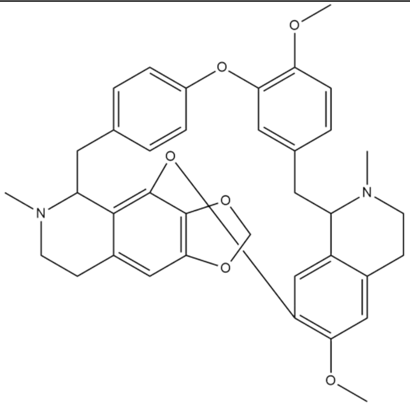
36.	Rhoifolin from *Litchi chinensis*	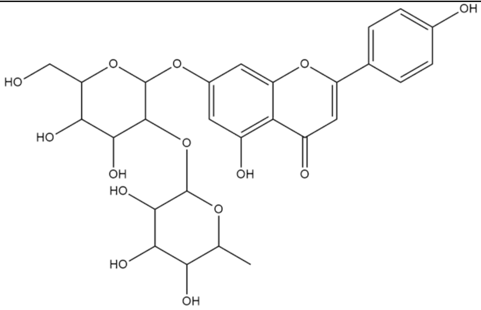

37.	Pectolinarin from *Litchi chinensis*	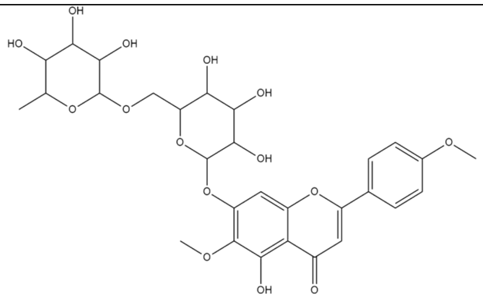
38.	Quercetin from *Pichia pastoris*	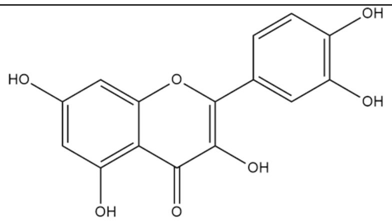
39.	Quercetin 3-β-D-glucoside	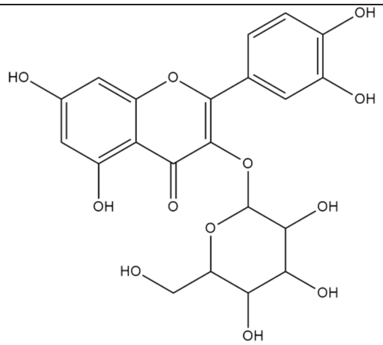
40.	Puerarin from *Pichia pastoris*	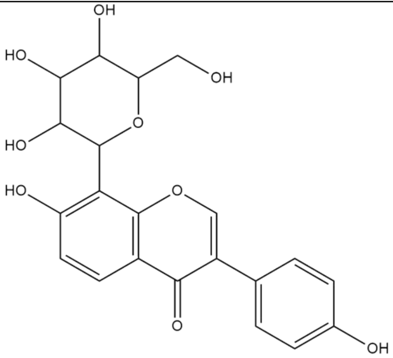
41.	Daidzein from *Pichia pastoris*	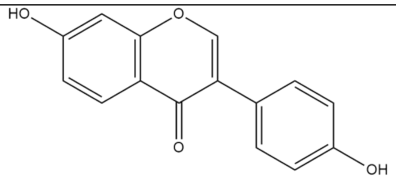

42.	Epigallocatechin from *Pichia pastoris*	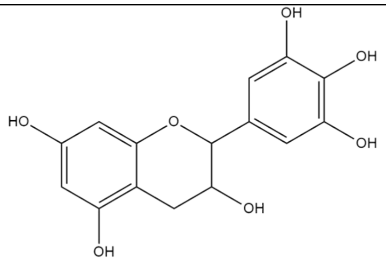
43.	Epigallocatechin gallate from *Pichia pastoris*	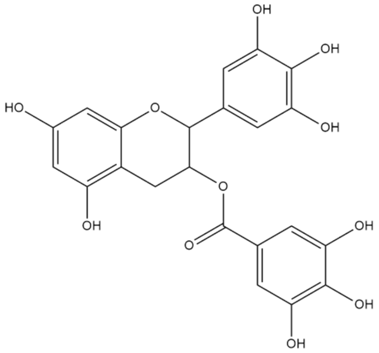
44.	Gallocatechin gallate from *Pichia pastoris*	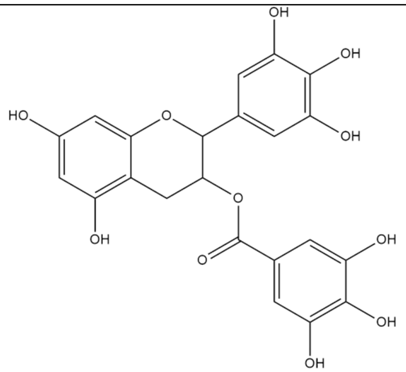
45.	Kaempferol	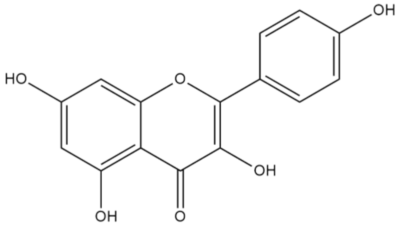
46.	Juglanin	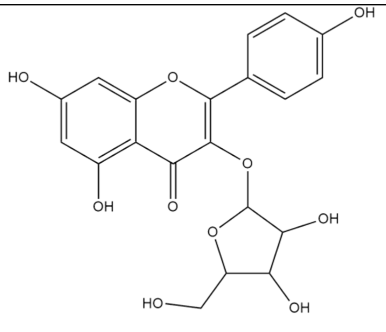

47.	Afzelin	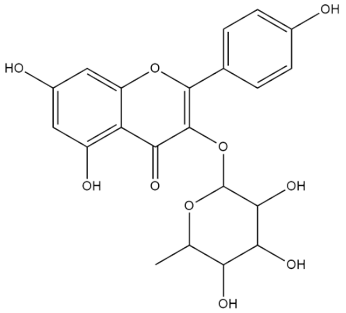
48.	Tiliroside	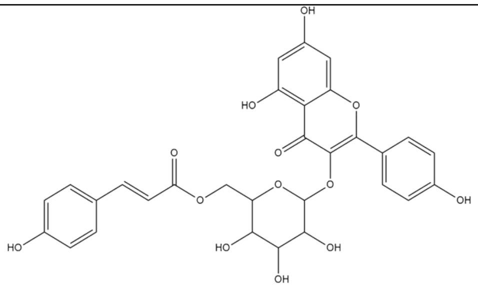
49.	Naringenin	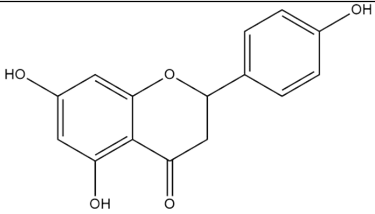
50.	Genistein	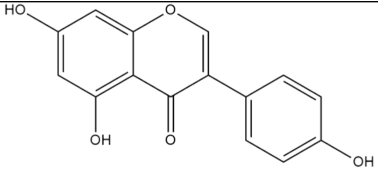
51.	Baicalin from *Scutellaria baicalensis*	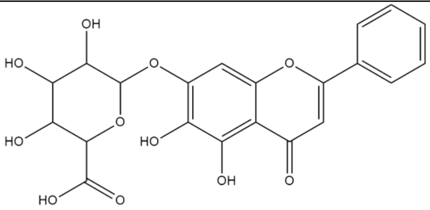
52.	Saikosaponin A	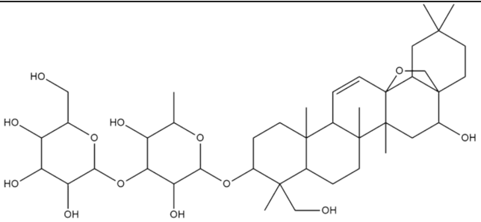

53.	Saikosaponin B2	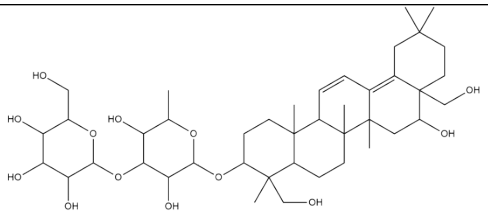
54.	Saikosaponin C	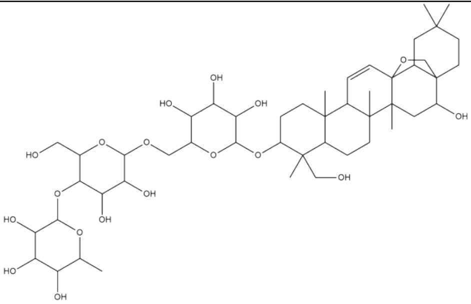
55.	Saikosaponin D	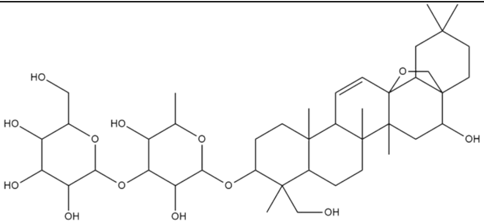
56.	Tetra-*O*-galloyl-β-D-glucose from *Galla chinensis* and *Veronicalina riifolia*	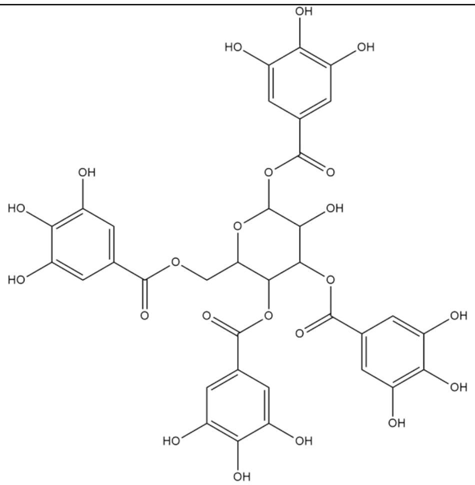
57.	Chlorogenic acid from *Flos lonicerae*	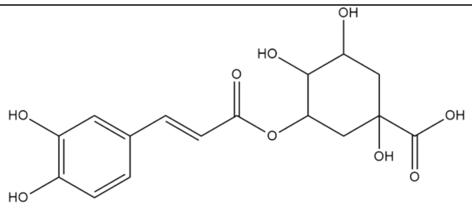

58.	Bavachinin from *Psoralea corylifolia*	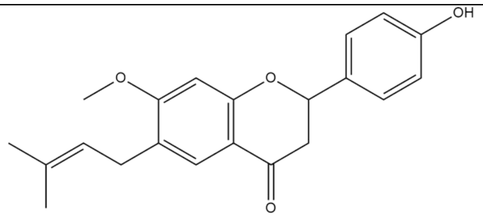
59.	Neobavaisoflavone from *Psoralea corylifolia*	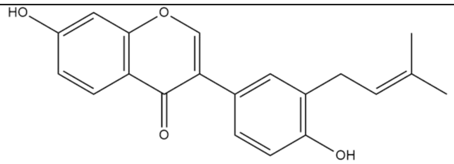
60.	Isobavachalcone from *Psoralea corylifolia*	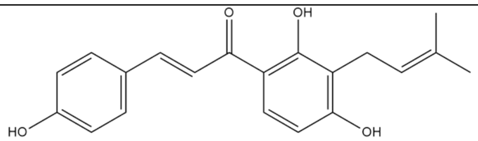
61.	4-*O*-Methylbavachalcone from *Psoralea corylifolia*	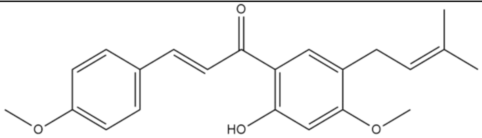
62.	Psoralidin from *Psoralea corylifolia*	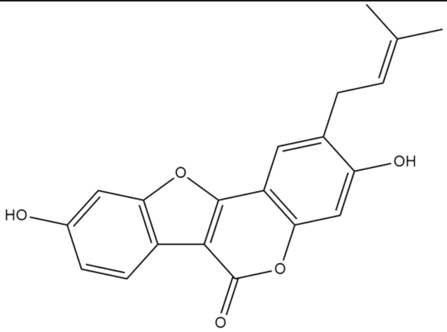
63.	Corylifol A from *Psoralea corylifolia*	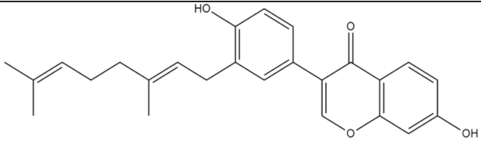
64.	Berbamine	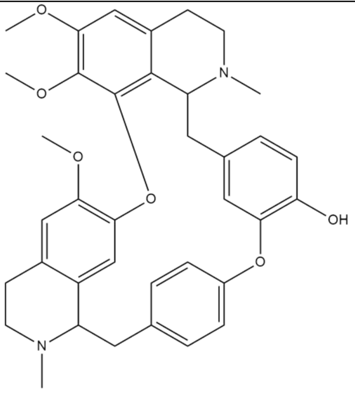

65.	Emetine	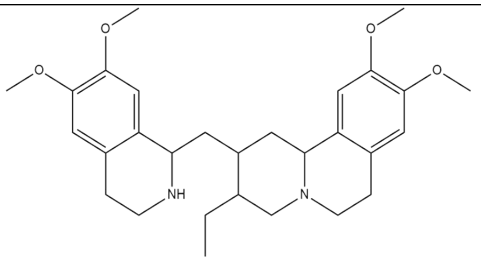
66.	Mycophenolate mofetil	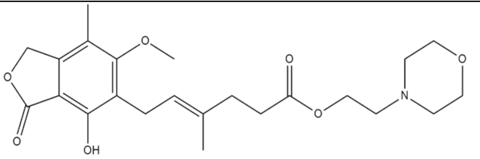
67.	Phenazopyridine	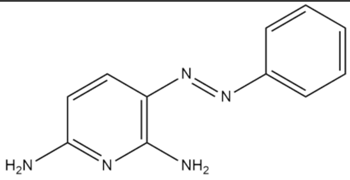
68.	Betulonic acid	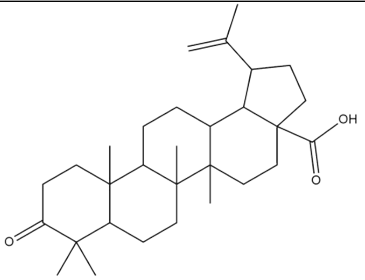
69.	Curcumin	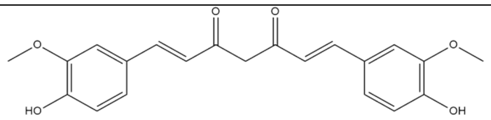
70.	Betulinic acid	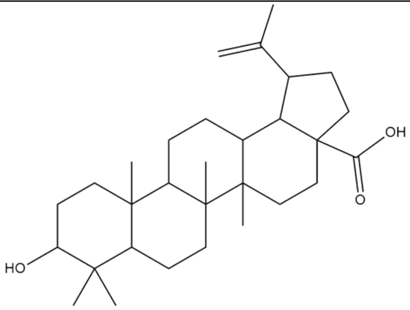

71.	3β,12-Diacetoxyabieta6,8,11,13-tetraene	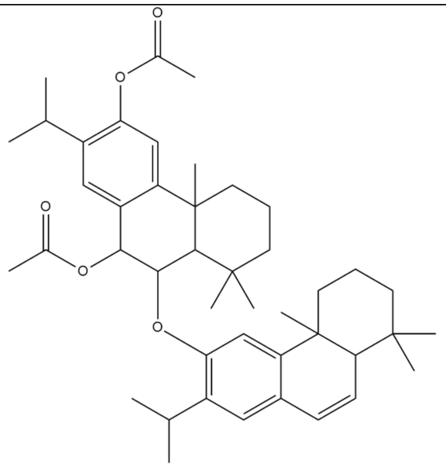
72.	Hinokinin	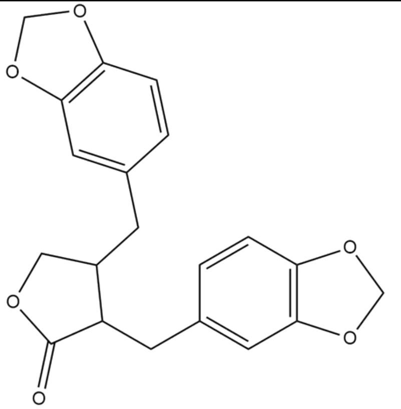
73.	Niclosamide	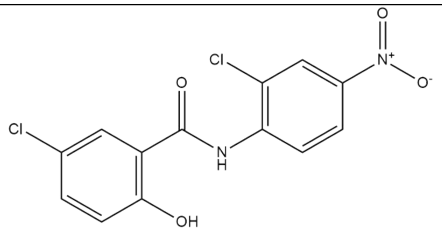
74.	Savinin	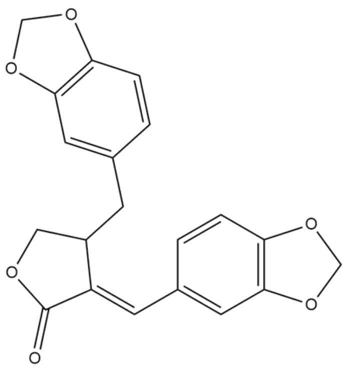

75.	Broussochalcone B1 from *Broussonetia papyrifera*	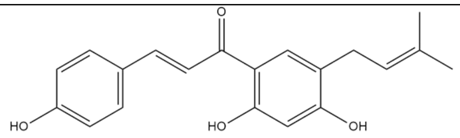
76.	Broussochalcone A from *Broussonetia papyrifera*	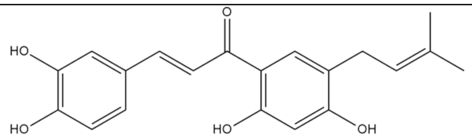
77.	4-Hydroxyisolonchocarpin from *Broussonetia papyrifera*	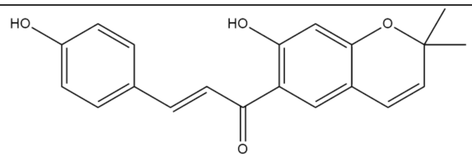
78.	Papyriflavonol A from *Broussonetia papyrifera*	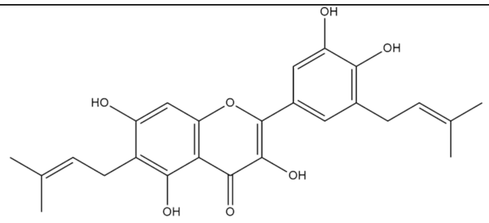
79.	3-(3-Methylbut-2-enyl)-3,4,7-trihydroxyflavane from *Broussonetia papyrifera*	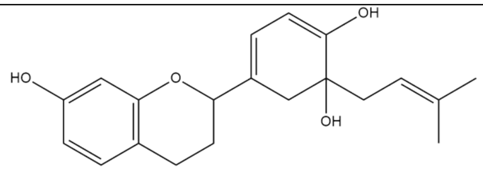
80.	Kazinol A from *Broussonetia papyrifera*	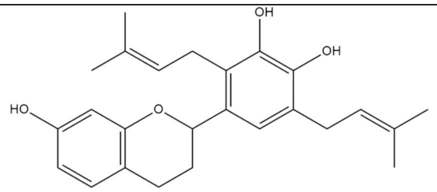
81.	Kazinol B from *Broussonetia papyrifera*	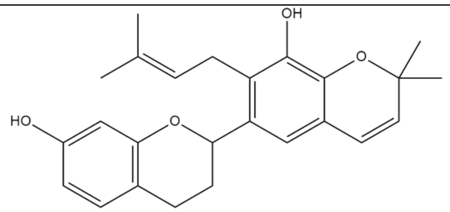
82.	Broussoflavan A from *Broussonetia papyrifera*	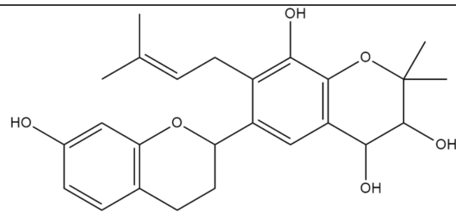

83.	Kazinol F from *Broussonetia papyrifera*	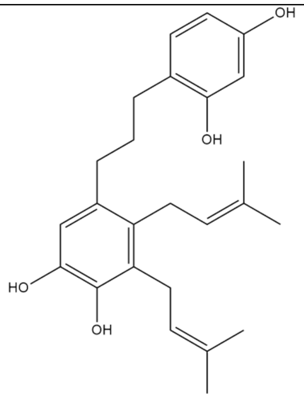
84.	Kazinol J from *Broussonetia papyrifera*	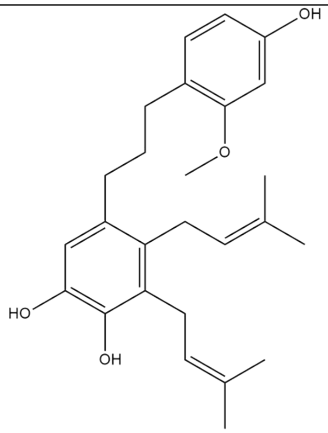
85.	Cinnamtannin B1 from Cinnamomi Cortex	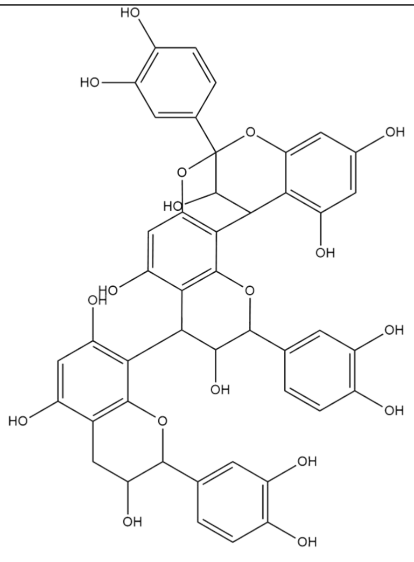

86.	Procyanidin A2 from Cinnamomi Cortex	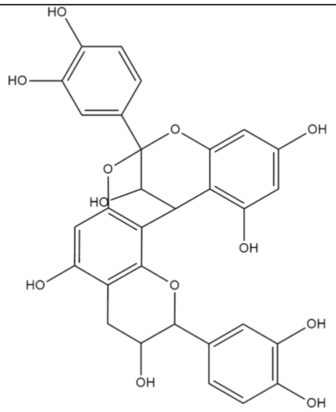
87.	Procyanidin B1 from Cinnamomi cortex	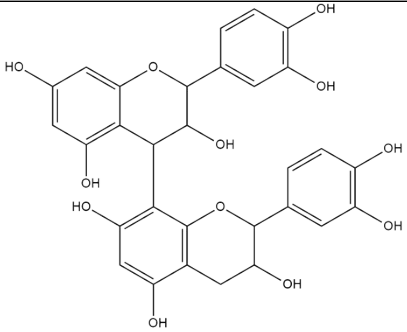
88.	Chrysin	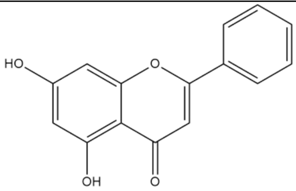
89.	Rhein from *Rheum officinale*	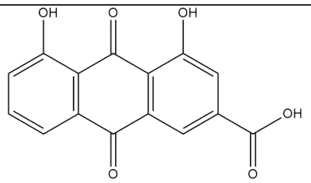
90.	Emodin derived from *Rheum officinale* and *Polygonum multiflorum*	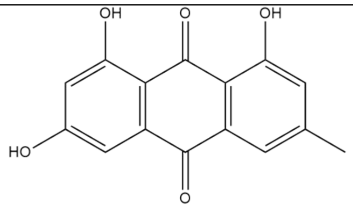
91.	Concanavalin A	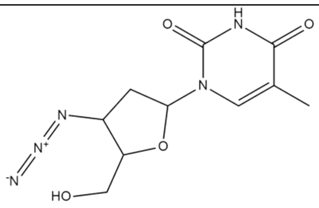

92.	Dieckol from *Ecklonia cava*	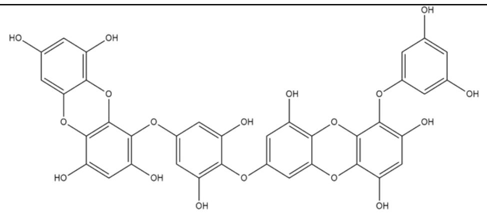
93.	Phloroglucinol from *Ecklonia cava*	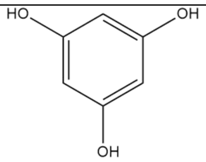
94.	Eckol from *Ecklonia cava*	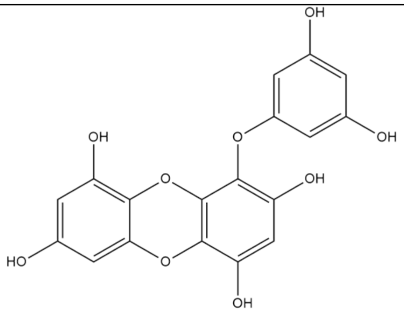
95.	7-Phloroeckol from *Ecklonia cava*	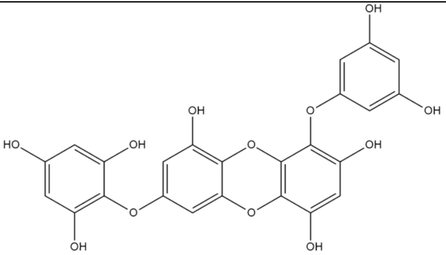
96.	Phlorofucofuroeckol from *Ecklonia cava*	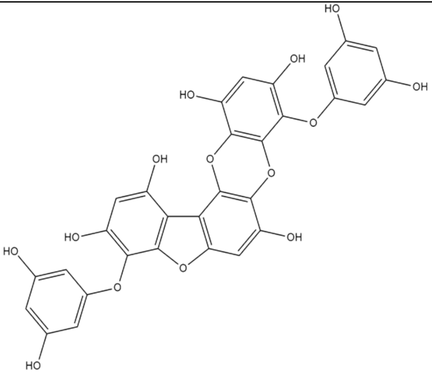
97.	Diplacone from *Paulownia tomentosa*	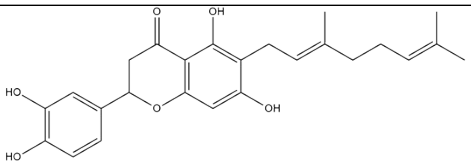

98.	Tomentin A from *Paulownia tomentosa*	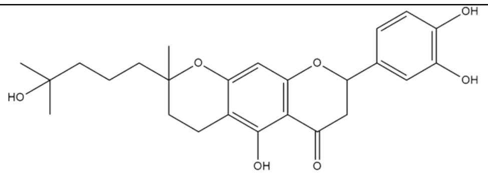
99.	Tomentin B from *Paulownia tomentosa*	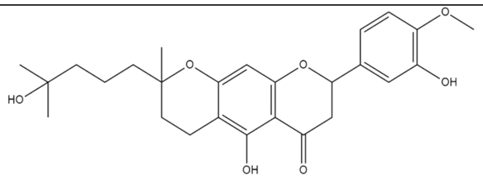
100.	Tomentin C from *Paulownia tomentosa*	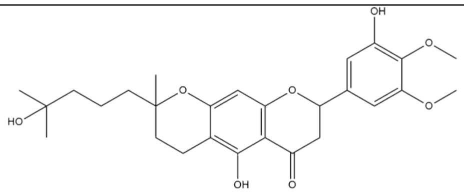
101.	Tomentin D from *Paulownia tomentosa*	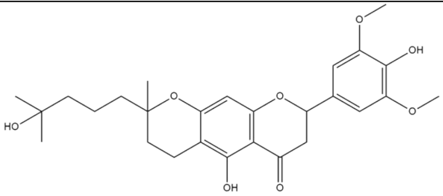
102.	Tomentin E from *Paulownia tomentosa*	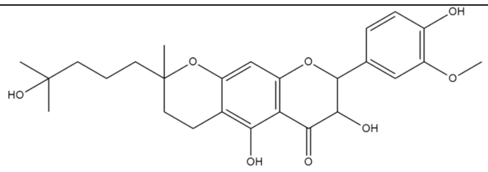
103.	3-O-methyldiplacol from *Paulownia tomentosa*	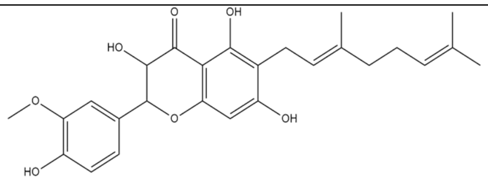
104.	4-O-methyldiplacol from *Paulownia tomentosa*	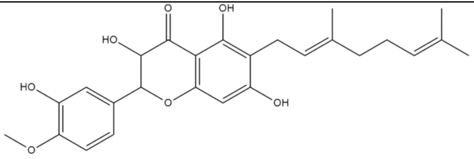
105.	3-O-Methyldiplacone from *Paulownia tomentosa*	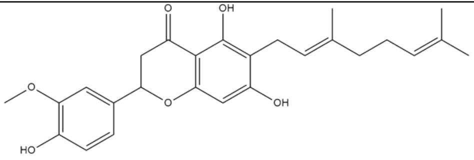
106.	4-O-Methyldiplacone from *Paulownia tomentosa*	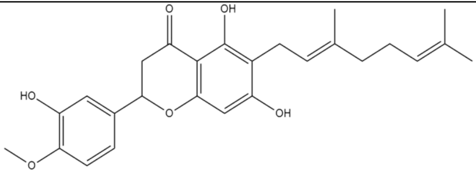

107.	Mimulone from *Paulownia tomentosa*	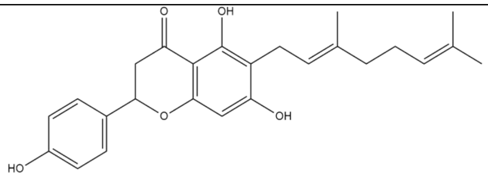
108.	6-Geranyl-4,5,7-trihydroxy-3,5-dimethoxyflavanone from *Paulownia tomentosa*	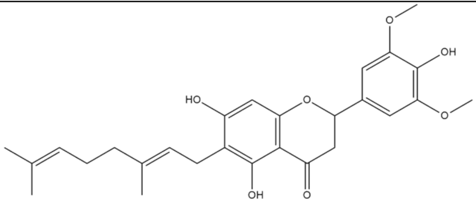
109.	Halitunal from *Halimeda tuna*	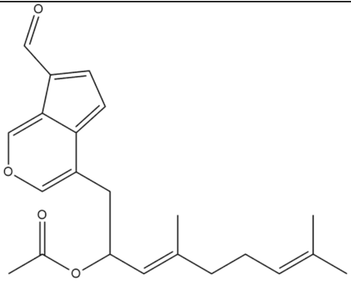
110.	Hexachlorophene	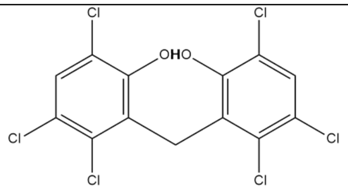
111.	Homoharringtonine	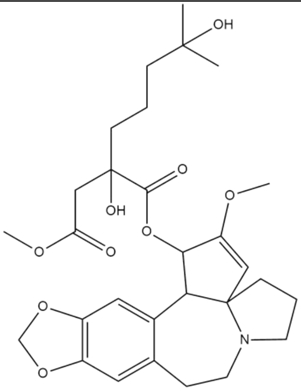
112.	Tannic acid from black tea	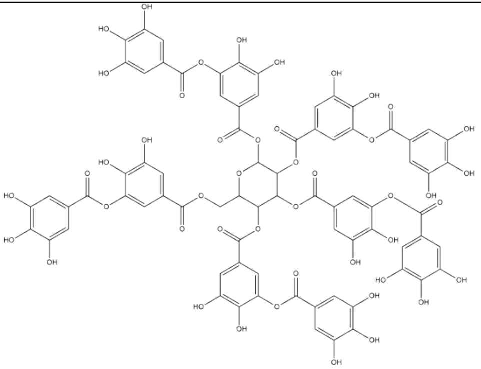

113.	Caffeine from black tea	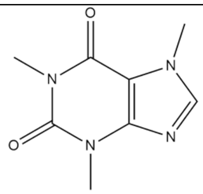
114.	Theophylline from black tea	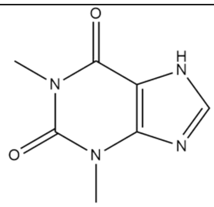
115.	Catechin from black tea	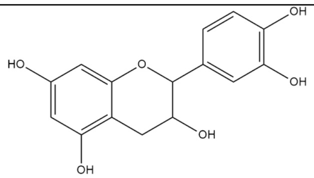
116.	Epicatechin from black tea	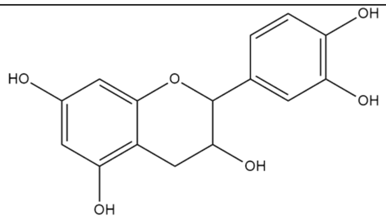
117.	Epicatechin gallate from black tea	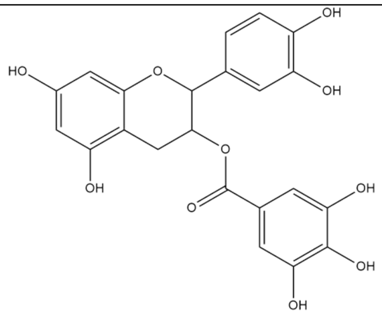
118.	Theaflavin from black tea	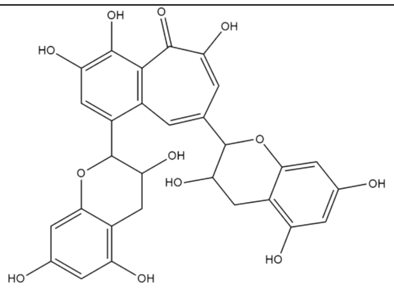

119.	Theaflavin-3-gallate from black tea	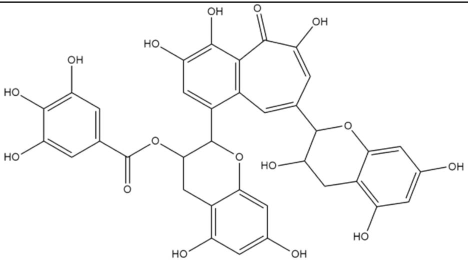
120.	Theaflavin-3,3-digallate from black tea	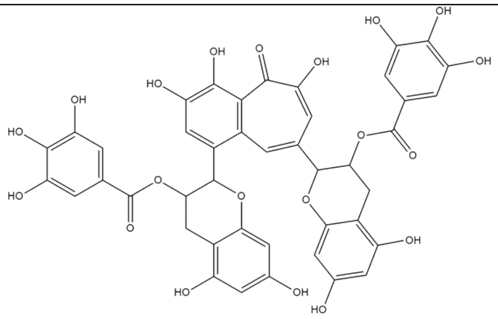
121.	3-Isotheaflavin-3-gallate from black tea	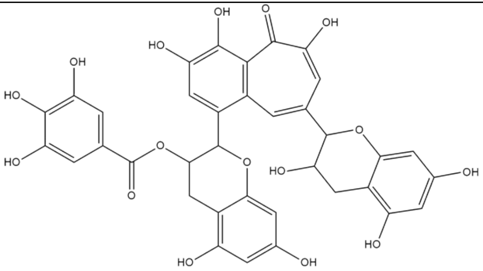
122.	7-Methoxycryptopleurine from *Tylophora indica*	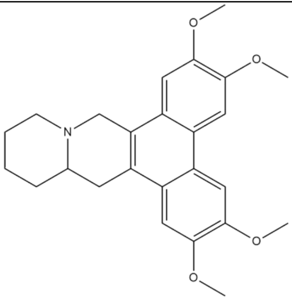
123.	Tylophorine from *Tylophora indica*	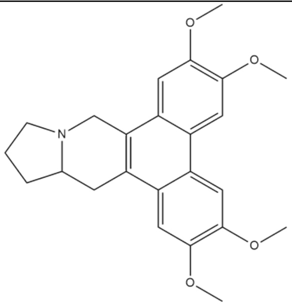

124.	Ouabain	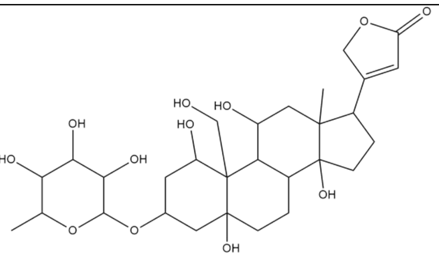
125.	Tanshinone IIA from *Salvia miltiorrhiza*	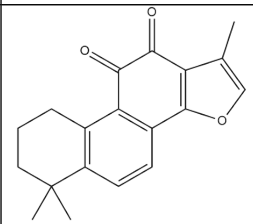
126.	Tanshinone IIB from *Salvia miltiorrhiza*	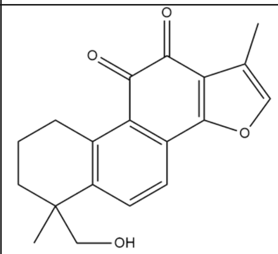
127.	Methyl tanshinonate from *Salvia miltiorrhiza*	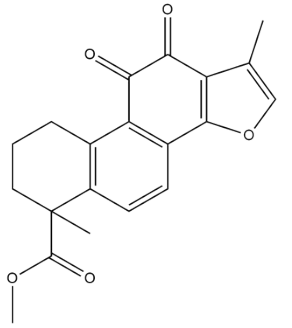
128.	Cryptotanshinone from *Salvia miltiorrhiza*	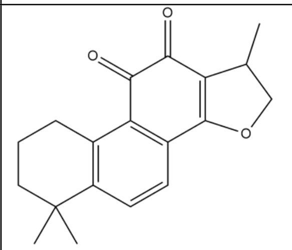
129.	Tanshinone I from *Salvia miltiorrhiza*	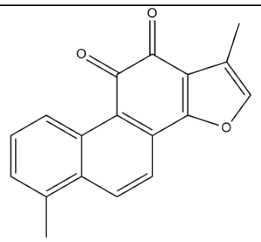

130.	Dihydrotanshinone I from *Salvia miltiorrhiza*	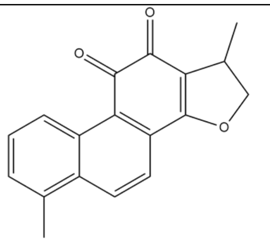
131.	Rosmariquinone from *Salvia miltiorrhiza*	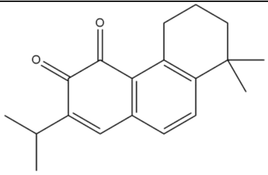
132.	Silvestrol	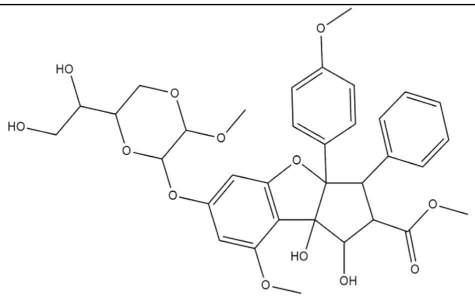

Suppl. Table 2.Hydrogen bond interaction parameters for selected compounds and M^{pro}s residues

S. No	Compound	Distance	Category	Type	From	From chemistry	To	To chemistry
1	Gallocatechin Gallate	2.18992	Hydrogen Bond	Water Hydrogen Bond; Conventional Hydrogen Bond	Gallocatechin gallate :H44	H-Donor	C:HOH201:O	H-Acceptor
2		2.9828	Hydrogen Bond	Conventional Hydrogen Bond	A:LEU167:HN1	H-Donor	Gallocatechin gallate :O7	H-Acceptor
3		2.21734	Hydrogen Bond	Conventional Hydrogen Bond	Gallocatechin Gallate :H48	H-Donor	A:THR190:O	H-Acceptor
5		1.91949	Hydrogen Bond	Conventional Hydrogen Bond	Gallocatechin Gallate :H50	H-Donor	A:LEU167:O	H-Acceptor

6		2.34856	Hydrogen Bond	Carbon Hydrogen Bond	A:MET165:HC	H-Donor	GallocatechinGallate :O7	H-Acceptor
7		2.68288	Hydrogen Bond	Carbon Hydrogen Bond	A:PRO168:HD2	H-Donor	GallocatechinGallate B1O8	H-Acceptor
8		2.50437	Hydrogen Bond	Carbon Hydrogen Bond	GallocatechinGallate :H35	H-Donor	A:GLN189:OE1	H-Acceptor
9	Cinnamatannin B1	1.854	Hydrogen Bond	Water Hydrogen Bond;Conventional Hydrogen Bond	C:HOH201:H1	H-Donor	Cinnamatannin B1:O9	H-Acceptor
10		1.98372	Hydrogen Bond	Conventional Hydrogen Bond	Cinnamatannin B1H86	H-Donor	A:LEU141:O	H-Acceptor
11		2.10048	Hydrogen Bond	Conventional Hydrogen Bond	Cinnamatannin B17:H88	H-Donor	A:GLN189:OE1	H-Acceptor
12		1.84203	Hydrogen	Conventional	Cinnamatannin	H-Donor	Cinnamatannin	H-Acceptor

			Bond	Hydrogen Bond	B1:H94		B1:O8	
13		1.98051	Hydrogen Bond	Conventional Hydrogen Bond	Cinnamatannin B1:H97	H-Donor	A:THR190:O	H-Acceptor
14		2.74535	Hydrogen Bond	Carbon Hydrogen Bond	A:GLY170:HA1	H-Donor	Cinnamatannin B1:O16	H-Acceptor
15		2.50807	Hydrogen Bond	Carbon Hydrogen Bond	Cinnamatannin B1H67	H-Donor	Cinnamatannin B1:O9	H-Acceptor
16	Remedesivir	2.0651	Hydrogen Bond	Water Hydrogen Bond;Conventional Hydrogen Bond	C:HOH201:H1	H-Donor	121304016:O9	H-Acceptor
17		2.55261	Hydrogen Bond	Water Hydrogen Bond;Carbon Hydrogen Bond	Remedesivir:H47	H-Donor	C:HOH201:O	H-Acceptor
18		2.40776	Hydrogen Bond	Carbon Hydrogen Bond	A:MET165:HA	H-Donor	Cinnamatannin B1:O7	H-Acceptor
19		2.44138	Hydrogen Bond	Carbon Hydrogen Bond	Remedesivir:H43	H-Donor	Cinnamatannin B1:N11	H-Acceptor

20		2.67508	Hydrogen Bond	Carbon Hydrogen Bond	Remedesivir:H44	H-Donor	A:HIS41:NE2	H-Acceptor

Suppl. Table 3.Pi-pi interaction parameters for selected compounds and M^{pro}s residues

S. No	Compound	Distance	Category	Type	From	From chemistry	To	To chemistry
1	Gallocatechin Gallate	5.08212	Other	Pi-Sulfur	A:MET165:SD	Sulfur	Gallocatechin Gallate	Pi-Orbitals
		5.26246	Hydrophobic	Alkyl	A:MET165	Alkyl	GallocatechinGallate	Alkyl
		4.94274	Hydrophobic	Pi-Alkyl	GallocatechinGallate	Pi-Orbitals	A:MET49	Alkyl
2	Cinnamatannin B1	5.95021	Other	Pi-Sulfur	A:MET165:SD	Sulfur	Cinnamatannin B1	Pi-Orbitals
		4.40528	Hydrophobic	Pi-Pi T-shaped	A:HIS41	Pi-Orbitals	Cinnamatannin B1	Pi-Orbitals
		4.64689	Hydrophobic	Amide-Pi Stacked	A:LEU167:C,O; PRO168:N	Amide	Cinnamatannin B1	Pi-Orbitals
		4.39324	Hydrophobic	Pi-Alkyl	Cinnamatannin B1	Pi-Orbitals	A:PRO168	Alkyl

3	Remedesivir	4.54362	Hydrophobic	Pi-Alkyl	Remedesivir	Pi-Orbitals	A:MET49	Alkyl
